# Reactive Oxygen Species (ROS)-Responsive Nanomedicine for Solving Ischemia-Reperfusion Injury

**DOI:** 10.3389/fchem.2020.00732

**Published:** 2020-08-21

**Authors:** Weiyu Chen, Deling Li

**Affiliations:** ^1^The Fourth Affiliated Hospital, Zhejiang University School of Medicine, Yiwu, China; ^2^Molecular Imaging Program at Stanford, Department of Radiology, Stanford University, Stanford, CA, United States; ^3^Department of Neurosurgery, Beijing Tiantan Hospital, Capital Medical University, Beijing, China

**Keywords:** bioresponsive nanomedicine, ischemia-reperfusion injury, reactive oxygen species, organ transplantation, nanoparticles

## Abstract

Ischemia-reperfusion injury (IRI) is a severe condition for most organs, which could occur in various tissues including brain, heart, liver, and kidney, etc. As one of the major hazards, reactive oxygen species (ROS) is excessively generated after IRI, which causes severe damage inside tissues and further induces the following injury via inflammatory response. However, current medical strategies could not thoroughly diagnose and prevent this disease, eventually leading to severe sequelae by missing the best time point for therapy. In the past decade, various nanoparticles that could selectively respond to ROS have been developed and applied in IRI. These advanced nanomedicines have shown efficient performance in detecting and treating a series of IRI (e.g., acute kidney injury, acute liver injury, and ischemic stroke, etc.), which are well-summarized in the current review. In addition, the nano-platforms (e.g., anti-IL-6 antibody, rapamycin, and hydrogen sulfide delivering nanoparticles, etc.) for preventing IRI during organ transplantation have also been included. Moreover, the development and challenges of ROS-responsive nanomedicine are systematically discussed for guiding the future direction.

## Introduction

Instead of restoring and saving tissues from hypoxic conditions, the reperfusion of blood would cause severe damage to those ischemic organs via oxidative stress, which is referred as ischemia-reperfusion injury (IRI). Normally, IRI is strongly associated with a series of diseases, such as the infraction of organs, including stroke, renal infraction, and trauma, etc. Notably, the concern of IRI often affects the best timing of reperfusion, the most effective therapy for treating ischemic disease, for example, the acute myocardial infarction (AMI) (Benjamin et al., 2017). Moreover, the acute myocardial IRI can lead to coronary heart disease (CHD) that accounts for over 366,800 deaths per year and more cases with disability (Benjamin et al., 2018).

In addition to the interruption of cellular homeostasis that could damage cells via apoptotic and autophagic pathways, ischemia-reperfusion would bring great oxidative stress and further injure organs via the generation of redox free radicals, for instance, reactive oxygen species (ROS) (Zhang et al., 2007). Although appropriate ROS is essential for intracellular signaling and immune response against pathogens, the excessive ROS can not be entirely scavenged by cellular antioxidants, which would mediate the severe damage on cells by destroying organelle, cell membrane, and nuclei DNA (Nita and Grzybowski, 2016). To protect injured tissues and prevent further damage of organs during transplantation, the administration of antioxidant reagents for scavenging ROS has been widely applied, such as edaravone (Fu et al., 2020). However, the usage of edaravone is generally accompanied by a series of side effects ranging from bruising to skin inflammation or rash, leading to many concerns and demands of new types of antioxidant reagents.

In the comparison of traditional drugs, the novel nanomedicine has been gifted with more functional features including high cargos loading, specific targeting, and *in-situ* theranostics (Chen et al., 2018a,b; Qiao et al., 2020). With these highly controllable functions, precision therapy could be achieved. As a representative, paclitaxel albumin-bound nanoparticles (Abraxane), the FDA-approved nanomedicine has been widely employed for fighting cancer. More importantly, various nanoparticles demonstrate excellent redox activity by trapping free radicals or self-oxidation (Ni et al., 2019). Notably, an increasing number of nano-antioxidants [e.g., Mo-based polyoxometalate nanoclusters (POM), framework nucleic acid (FNA), hydrophobic ceria nanoparticles (NPs), etc.] were recruited as ROS scavenger in dealing with various diseases associate with IRI (e.g., acute kidney injury, acute liver injury, and ischemic stroke, etc.). Here, those advanced ROS-responsive nanoparticles along with antioxidants delivering nano-platforms, have been summarized in the current review to highlight the recent innovation of nanomedicine in dealing with IRI associated diseases. Moreover, the perspective of future development and facing challenges are systemically discussed.

## IRI and its Threats

Organs will become ischemic if related vasculars were blockaged, which could induce irreservable damage inside the organs. However, the acute reperfusion for ischemics would cause IRI in some patients, which has been widely observed in various diseases including acute coronary syndrome, hepatic/renal acute injury and organ transplantation (Varadarajan et al., 2004). Eventrually, this unexpected complication, IRI will directly lead to a series pathological changes that manifests in brain edema, infarct progression, hemorrhagic transformation, and worsening neurologic system (Zhai et al., 2013; Prabhakaran et al., 2015). More importantly, the severe IRI may further induce systemic diseases including the inflammatory response syndrome (SIRS) and multiple organ dysfunction syndrome (MODS), and increase the mortality of patients, especially for those from the intensive care unit (Cryer, 2000).

## General Strategies for Dealing IRI

How to deal with the IRI is of clinical importance, therefore arousing the interests of diverse scientific communities. From the aspect of clinical view, three key factors have been focused on improving the efficiency and safety of reperfusion treatment, including developing different generation thrombectomy devices, lowering the time to reperfusion and applying potential imaging techniques for enrolling suitable patients (Prabhakaran et al., 2015). However, the reality of the synergistic role of various biological mechanisms in IRI made it challenging to prevent or treat. Indeed, some medications with predicted benefits to IRI have not been proved with a definite role in prevention or treatment, likely vitamin C (Hill et al., 2019), morphine (Le Corvoisier et al., 2018), etc. In the field of liver transplantation, nearly all randomized clinical trials have ultimately failed to ameliorate liver IRI in patients (Zhai et al., 2013). Therefore, innovative strategies with smart features for targeting local environmental factor, such as ROS would be an exciting direction for IRI treatment in the future.

## Nanomedicine Applied in IRI Therapy

Ischemia could be induced via physical conditions (e.g., elevated blood pressure and diabetes, etc.) or damage, which can further trigger following IRI in various organs, ranging from cerebral to renal system. Notably, the elevated ROS after reperfusion mainly contributes to cellular and tissue damage. Recently, a series of novel nanomedicine (e.g., POM and Ceria NPs, etc.) has been designed for treating affected tissues via the smartly ROS-responsive scavenge and imaging of redox in the ischemic area (Table 1).

**Table 1:** The representatives of novel nanomedicine for treating various IRI via ROS savaging.

**Application**	**Type of materials**	**Nanomedicine**	**Size (nm)**	**Mechanism**	**Investigation**	**References**
Cerebral IRI	FNA	aC5a-FNA	10	Antioxidant (FNA)/C5a-blocking	Protection for cerebral I/R	Li et al., 2019b
Cerebral IRI	Polyoxometalate	POM	~1	Antioxidant	Protection for cerebral I/R	Li et al., 2019a
Cerebral IRI	Polymer	t-PA@iRNP	48 ± 2	Antioxidant (LMW nitroxide)/Thrombolysis	Protection for Middle cerebral artery occlusion	Mei et al., 2019
Cardiac IRI	Polymer	PEG-b-PPS	~100	Antioxidant (ginsenoside Rg3)	Protection for Myocardial IRI	Li et al., 2020
Cardiac IRI	Polymer	CLP NPs	150 ± 18	Enhanced FL signal via oxidative stress	IVIS imaging of Myocardial IRI	Ziegler et al., 2019
Cardiac IRI	Polymer	MCTD-NPs	95	Antioxidant (TPP)	Protection for Myocardial IRI	Cheng et al., 2019
Hepatic IRI	Ceria	Ceria NPs	4.48 ± 0.8	Antioxidant	Protection for Partial hepatic IRI	Ni et al., 2019
Hepatic IRI	Ceria	CeO_2_	10–30	Antioxidant	Protection for Partial hepatic IRI	Manne et al., 2017
Hepatic IRI	Polymer	BRNPs	100	Antioxidant (Bilirubin)	Protection for Partial hepatic IRI	Kim et al., 2017
Hepatic IRI	Polymeric	PVO	550	Antioxidant (peroxalate esters)/Gas generation	Protection for and US imaging Partial hepatic IRI	Kang et al., 2016
Renal IRI	Carbon QDs	SeCQDs	40	Antioxidant (doping selenium)	Protection for Glycerol/Cisplatin-induced AKI Models	Rosenkrans et al., 2020
Renal IRI	FNA	DONs	90, 120, 400	Antioxidant	Protection for Glycerol-induced AKI Model	Jiang et al., 2018
Renal IRI	Melanin	MMPP NPs	4.5	Antioxidant	Protection for Glycerol-induced AKI Model	Sun et al., 2019
Cardiac IRI	Silica	DATS-MIONs	230 ± 35	H_2_S delivery (DATS)	Protection for Myocardial IRI	Wang et al., 2019
Organ transplant	Polymer	Anti–IL-6–PLGA-NPs	100	Anti-IL-6 delivery	Protection for Heart allograft	Solhjou et al., 2017
Organ transplant	Micelles	TRaM	15.3 ± 2.3	Rapamycin delivery	Protection for Aortic interposition allografts	Zhu et al., 2018

*FNA, Framework nucleic acid; DONs, DNA origami nanostructures; FL, Fluorescence; TPP, Triphenylphosphine; LMW, Low molecular weight; IVIS, in vivo fluorescence imaging system; AKI, Acute kidney injury; PEG, Poly ethylene glycoland; PPS, Poly propylene sulfide*.

## Nanomedicine for Cerebral IRI

Cerebral ischemia that generally caused by stroke is associated with high mortality and disability rate. More importantly, ROS produced by cerebral ischemia-reperfusion can further damage brain tissues via neurodegeneration. To overcome this severe disease, various of nanomaterials including polymer NPs (Reddy and Labhasetwar, 2009; Petro et al., 2016; Ghosh et al., 2017; Mei et al., 2019; Mukherjee et al., 2019), ceria NPs (Zhang et al., 2018), polyoxometalate (POM) nanoclusters (Li et al., 2019a), and Framework Nucleic Acids (Li et al., 2019b) have been applied as protective agents, indicating the high efficiency in saving cerebral tissue via ROS scavenging. Notably, the high biocompatibility allows the polymer to be widely recruited for delivering antioxidant reagents [i.e., superoxide dismutase (Reddy and Labhasetwar, 2009), antioxidants catalas (Petro et al., 2016), and curcumin (Mukherjee et al., 2019), etc]. Besides, a dual functional polymer NPs, t-PA@iRNP was successfully employed to induce thrombolytic and antioxidant therapies in cerebral tissue (Mei et al., 2019). With the encapsulation of tissue plasminogen activator (t-PA) and conjugation of 4-amino-2,2,6,6-tetramethylpiperidine-1-oxyl (4-amino-TEMPO), self-assemble t-PA@iRNP (~50 nm) demonstrated an acidic-triggered (pH = 6.2) thrombolytic activity and a significant decrease of ROS production in middle cerebral artery occlusion (MCAO) model mice. More importantly, the antioxidant effect of t-PA@iRNP efficiently avoided the subarachnoid hemorrhage induced by t-PA, releasing a great potential of this dual therapy via synergistic effect.

**Figure 1:**
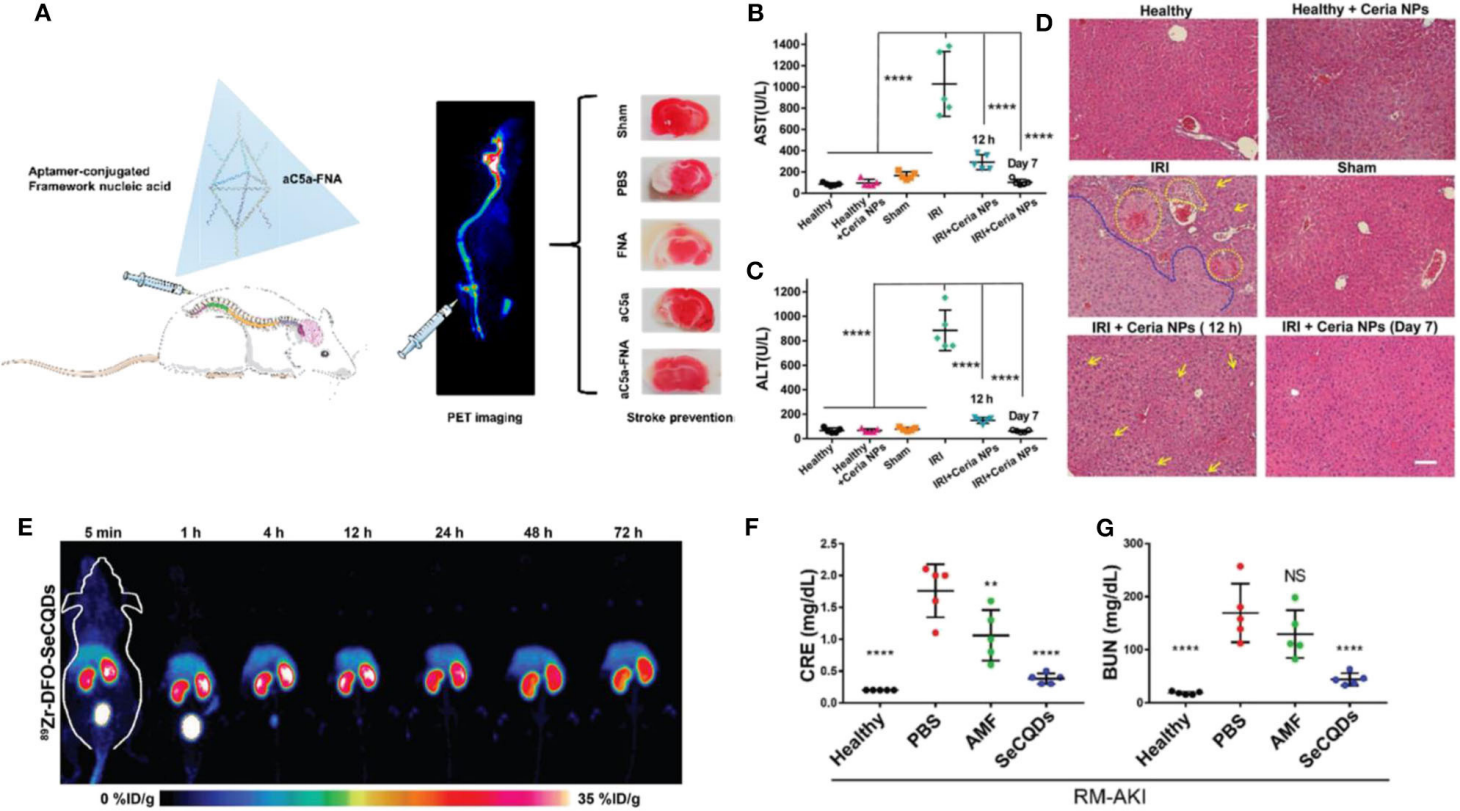
Advanced ROS-responsive nanomedicine for various IRI therapy. **(A)** The aptamer-conjugated FNA for cerebral IRI therapy. Reproduced with permission from Li et al. (2019b). Copyright 2019, American Chemical Society. The therapeutic effects mediated by Ceria NPs in liver IRI. The level of AST **(B)** and ALT **(C)** in serum after different treatments (****p < 0.0001); **(D)** The HandE staining of liver harvested from different groups. The damage of hepatic cells (i.e., hemorrhage, cytolysis, and necrosis) and severely damaged tissue areas are labeled by yellow and blue dash lines, respectively. Lipid droplets formed are indicated by yellow arrows. Scale bar: 100 μm. Reproduced with permission from Ni et al. (2019). Copyright 2019, Wiley. The application of SeCQDs in treating AKI for protecting kidney from renal IRI. **(E)** The time-dependent bio-distribution of ^89^Zr-DFO-SeCQDs in healthy ICR mice via MIP-PET images; The CRE **(F)** and BUN **(G)** concentration in serum among groups after various treatments (**P < 0.01, ****P < 0.0001, NS: no significant difference). Reproduced with permission from Rosenkrans et al. (2020). Copyright 2020, Wiley.

In addition, novel bio-responsive nanomaterials containing the different status of metal ion (i.e., Mo^5+^ and Mo^6+^) or oxygen-sensitive bio-structure (i.e., DNA) showed excellent capabilities as antioxidants (Li et al., 2019a,b). For instance, a POM nanocluster was developed and employed as an ROS-responsive agent in treating rats with cerebral I/R injury (Li et al., 2019a). After intrathecal injection, POM could efficiently accumulate in the brain and reduce the oxidative pressure, eventually suppressing the apoptosis of cerebral cells, edema, and infarct volume within the brain (for about 40~50% decrease). Accompanied by IRI, excessive inflammation plays as another key factor for neuronal damage. Recently, a framework nucleic acids (FNA) conjugating with anti-C5a aptamer (aC5a) was applied in the therapy against IRI in the brain (Figure 1A) (Li et al., 2019b). More specifically, the aC5a-FNA could effectively reduce the C5a receptor on polymorphonuclear neutrophils (PMNs) and microglia, eventually bringing down the chemotaxis induced by C5a. Meanwhile, the concentrations of C5a (within plasma and penumbra) and detrimental ROS in cerebral issues were well-scavenged by aC5a-FNA administration, which efficiently protects brain cells from IRI, with a much smaller infarct volume of 17.0 ± 5.6% in comparison with that in control group (41.6 ± 7.1%).

## Nanomedicine for Cardiac IRI

Among the leading causes of death, cardiovascular disease (CVD), especially the myocardial infarction, is extremely dangerous for any patient. As the biggest challenge, the prevention of IRI greatly affects the outcome of therapy for myocardial infarction. To date, several ROS-responsive nanomaterials have been recruited in cardiac IRI therapy via the delivery of antioxidants (i.e., resveratrol and Ginsenoside Rg3, etc.) (Bae et al., 2016; Cheng et al., 2019; Li et al., 2020). This year, a self-assemble nanoparticle poly ethylene glycol-b-poly propylene sulfide (PEG-b-PPS) that smartly responses to ROS was successfully synthesized and used for ginsenoside Rg3 (Rg3) delivery (Li et al., 2020). PEG-b-PPS-Rg3 exhibited a ROS-dependent release of Rg3 (80% release of Rg3 within 1 day when 1.0 mM ROS was presented) and induced antioxidative stress effects by a strong interaction with FoxO3a (−6.0 kcal/mol). More importantly, the administration of PEG-b-PPS-Rg3 successfully increased the survival rate of I/R rat (90%) in comparison with the group injected with Free Rg3 (40%), indicating its high efficiency in responding to and scavenging cardiac-generated ROS. It is notable that precision targeting may increase therapeutic efficiency. To achieve that, Cheng et al. further modified Szeto-Schiller (SS)-31 and ischemic myocardium targeted peptide (IMTP) for preventing myocardial IRI via mitochondria-targeted antioxidant effect (Cheng et al., 2019). As-prepared MCTD-NPs specifically deliver resveratrol into cardiac tissues, reducing sizes of the infraction and protecting cardiomyocyte from ROS damage.

Notably, the elevated intracellular Ca^2+^ is strongly associated with the ROS generation in cardiac cells, which provides another therapeutic strategy via the intervention of Ca^2+^ influx (via anti-L-Type Ca^2+^ Peptide and CaMKII inhibitor) (Hardy et al., 2015; Wongrakpanich et al., 2017). However, the efficiency of channel blockage is relatively lower. Thus, the combination of antioxidant (i.e., curcumin) reagents and mitochondria-targeting functionalization (i.e., TPP) are required for improving the therapeutic effect.

Besides, several ROS-responsive nano-platforms were also developed for monitoring the status of IRI during the therapy (Yang et al., 2016; Ziegler et al., 2019). Specifically, Peter Lab developed CLP nanoparticles that could smartly light up the ROS-generated myocardium area after ischemic/reperfusion (Ziegler et al., 2019). The presentation of ROS could restore the fluorescent intensity of Ce6 that was attenuated by the aggregation-induced fluorescence quenching. By this strategy, damaged cardiac areas could be real-time detected and monitored throughout the first day after reperfusion.

## Nanomedicine for Hepatic IRI

Liver, one of the major organs has played a key role in metabolisms and been involved in various functions, which demands a lot of blood supply. Hepatic ischemia and IRI, a serious complication caused by liver diseases or transplantation would directly trigger irreversible damage in liver tissue. In comparison with other organs, especially the brain, the liver is able to actively “uptake” foreign agents from blood, which allows a series of therapeutic agents to accumulate inside. Thus, antioxidant gene delivery (i.e., expression genes of extracellular superoxide dismutase, catalase or TLR-4 siRNA) (He et al., 2006; Jiang et al., 2011), antioxidant prodrug (i.e., BRAP, etc.) (Lee et al., 2015), and nanoparticles (CeO_2_ and platinum NP_S_, etc.) (Katsumi et al., 2014; Kang et al., 2016; Li et al., 2016; Kim et al., 2017; Manne et al., 2017; Ni et al., 2019) were recruited in treating herpetic IRI. For example, a nanoparticle was designed to release p-hydroxybenzyl alcohol (HBA) when H_2_O_2_ or LPS was presented, which could remove ROS and decrease the level of pro-inflammation factors (i.e., NO and TNF-α) (Lee et al., 2015). Moreover, Kang et al. (2016) developed smartly bio-responsive nanoparticles that could provide ultrasound (US) imaging signal during H_2_O_2_ scavenge. As-prepared PVO nanoparticles could quickly react with ROS and generate CO_2_ in liver tissue with IRI, offering detectable US signals as early as 25 min post injection.

Additionally, metal nanomaterials exhibiting high reducibility were used as therapeutic agents for hepatic IRI. Among all, CeO_2_ NP_S_ demonstrated high biocompatibility and efficiency. More specifically, a uniform-sized ceria NPs (~4.5 nm) was synthesized, containing two valences of Ce (32.01% of Ce^3+^ and 67.99% of Ce^4+^) (Ni et al., 2019). Administration of CeO_2_ NP_S_ greatly attenuated the IRI in liver tissue as early as 12 h post reperfusion (Figures 1B–D). It is crucial that the ROS scavenge mediated by CeO_2_ NP_S_ efficiently inhibits the pro-inflammation cytokine secretion (i.e., IL-12, IL-1, and TNF-α, etc.) and an influx of immune cells (i.e., monocyte and neutrophils). Meanwhile, CeO_2_ NP_S_ also illustrated a high quality in preventing intestinal cells from ROS-based IRI (Gubernatorova et al., 2017).

## Nanomedicine for Renal IRI

Acute kidney injury (AKI) that is triggered by diseases or physical damage could lead to renal disorder/failure. In addition to general therapies like dialysis, ROS-responsive nanomedicine may provide another approach for AKI treatment. Mainly, the functional incapacitation of kidney will directly result in passive accumulation of “waste” inside, including nanomedicine injected. By taking this shortcut, advanced nanomaterials with additional functions such as acid-activated (Yoshitomi et al., 2011) or magnetic resonance imaging (MRI) (Sun et al., 2019) were designed for dealing AKI via ROS reduction. Moreover, Minami et al. also synthesized a nanomedicine (e.g., APP-103) that could effectively accumulate in non-functional kidneys and protect them from IRI during AKI or transplantation (Minami et al., 2020).

In comparison, Cai's lab successfully developed two nanomedicines specifically targeting to the kidney. Both nanoparticles, SeCQDs and DONs (DNA origami nanostructures) predominantly accumulated in the health murine kidney with ~10 and ~27%ID/g, respectively, while the kidney after AKI further enhanced the accumulation of nanoparticles (Figure 1E) (Jiang et al., 2018; Rosenkrans et al., 2020). With oxidant-sensitive structures (e.g., doped selenium and nuclei acid), SeCQDs and DONs efficiently removed excessive ROS from renal cells (Figures 1F,G).

## Alternative Strategies for IRI Therapy and Organ Transplantation

In addition to an active reduction of cellular ROS, cytoprotective reagents such as hydrogen sulfide (H_2_S) are also applied as an anti-inflammation agent in various medical situations associated with ischemic diseases. It is notable that various smart nanomedicines have been prepared for H_2_S delivery, showing promising efficiency (Chen et al., 2019). More specifically, as-designed mesoporous iron oxide and silica nanoparticles could effectively carry and deliver H_2_S donor diallyl trisulfide (DATS) to ischemic organs (i.e., heart and aortic graft endothelium), eventually reducing the flowing injury caused by reperfusion (Wang et al., 2016, 2019).

Meanwhile, nano-platforms conjugating with immune-modulators could mediate desirable protection for injured or transplanted organs as well. For instance, the modification of antibodies on PLGA NPs successfully moderated the chronic rejection of transplanted heart by targeting IL-6 secreted by allograft-derived dendritic cells (ADDCs) (Solhjou et al., 2017). Furthermore, the endothelial cell inflammation and pro-inflammatory cytokines secretion following tracheal or aortic allografts could be greatly attenuated by the pre-treatment of rapamycin (Nadig et al., 2015; Zhu et al., 2018).

## Conclusion/Future Directions

ROS generated by ischemic reperfusion plays a crucial role in the following tissue injury. In other words, this also provides an efficient approach for dealing and preventing IRI via the reduction of ROS. Novel nanoparticles that smartly respond or consume ROS have been designed and proved for their promising performance. However, several directions and challenges should be aware for the future design of ROS-responsive nanomedicine. (a) A combination of multiple functions (i.e., imaging and therapy) in single nanomedicine is highly recommended for monitoring and treating IRI diseases; (b) Desirable IRI therapy may be achieved via dual therapy of ROS scavenge and immune-repress (i.e., H_2_S delivery); (c) Although alternative strategies, such as antioxidant gene delivery, may provide another direction for IRI therapy, the quick response and antioxidant effects are still the key issues for the future design of ROS-responsive nano-platforms; (d) Tissue-depended design is strongly recommended as well. For example, the antioxidant capability is more important than tissue targeting in AKI model (e.g., passive accumulation in AKI); (e) Sub-organ/cell-targeting for IRI therapy is highly suggested (i.e., antibody-induced cell targeting). Specifically, the most of antioxidant nanoparticles would be internalized by Kupffer cells during the liver IRI, which may lower the therapeutic effect of nanomedicine in major hepatic cells such as liver sinusoidal endothelial cells; (f) The antioxidant efficiency and biocompatibility should be balanced. Thus, advanced ROS-responsive nanomedicine have shown high-performance in dealing with various IRI, which exhibits a great potential in clinical translation, especially organ transplantation.

## Author Contributions

WC planned and wrote this review with the help of DL. All authors contributed to the article and approved the submitted version.

## Conflict of Interest

The authors declare that the research was conducted in the absence of any commercial or financial relationships that could be construed as a potential conflict of interest. The handling editor declared a past co-authorship with one of the authors WC.
